# Efficacy of Vemurafenib in Patients With Non–Small-Cell Lung Cancer With *BRAF* V600 Mutation: An Open-Label, Single-Arm Cohort of the Histology-Independent VE-BASKET Study

**DOI:** 10.1200/PO.18.00266

**Published:** 2019-06-27

**Authors:** Vivek Subbiah, Radj Gervais, Gregory Riely, Antoine Hollebecque, Jean-Yves Blay, Enriqueta Felip, Martin Schuler, Anthony Gonçalves, Antonio Italiano, Vicki Keedy, Ian Chau, Igor Puzanov, Noopur S. Raje, Funda Meric-Bernstam, Martina Makrutzki, Todd Riehl, Bethany Pitcher, Jose Baselga, David M. Hyman

**Affiliations:** ^1^ University of Texas MD Anderson Cancer Center, Houston, TX; ^2^ Centre François Baclesse, Caen, France; ^3^ Memorial Sloan Kettering Cancer Center, New York, NY; ^4^ Institut Gustave Roussy, Villejuif, France; ^5^ Centre Léon Bérard, Lyon, France; ^6^ Vall d’Hebron University Hospital and Vall d’Hebron Institute of Oncology, Barcelona, Spain; ^7^ University Hospital Essen, Essen, Germany; ^8^ Aix-Marseille Université, Marseille, France; ^9^ Institut Bergonie, Bordeaux, France; ^10^ Vanderbilt University, Nashville, TN; ^11^ The Royal Marsden NHS Foundation Trust, London, United Kingdom; ^12^ Roswell Park Cancer Institute, Buffalo, NY; ^13^ Massachusetts General Hospital, Boston, MA; ^14^ F. Hoffmann-La Roche, Basel, Switzerland; ^15^ Genentech, South San Francisco, CA; ^16^ F. Hoffmann-La Roche, Mississauga, Ontario, Canada; ^17^ Weill Cornell Medical College, New York, NY

## Abstract

**PURPOSE:**

To study whether *BRAF* V600 mutations in non–small-cell lung cancer (NSCLC) may indicate sensitivity to the BRAF inhibitor vemurafenib, we included a cohort of patients with NSCLC in the vemurafenib basket (VE-BASKET) study. On the basis of observed early clinical activity, we expanded the cohort of patients with NSCLC. We present results from this cohort.

**METHODS:**

This open-label, histology-independent, phase II study included six prespecified cohorts, including patients with NSCLC, and a seventh all-comers cohort. Patients received vemurafenib (960 mg two times per day) until disease progression or unacceptable toxicity. The primary end point of the final analysis was objective response rate (Response Evaluation Criteria in Solid Tumors, version 1.1). Secondary end points included progression-free survival, overall survival, and safety. Because the prespecified clinical benefit endpoint was met in the initial NSCLC cohort, the cohort was expanded.

**RESULTS:**

Sixty-two patients with *BRAF* V600–mutant NSCLC were enrolled and treated: 13% (n = 8) had received no prior systemic therapy, and 87% (n = 54) had received prior therapies. The objective response rate was 37.1% (95% CI, 25.2% to 50.3%) overall, 37.5% (95% CI, 8.5% to 75.5%) in previously untreated patients, and 37.0% (24.3% to 51.3%) in previously treated patients. Median progression-free survival was 6.5 months (95% CI, 5.2 to 9.0 months), and median overall survival was 15.4 months (95% CI, 9.6 to 22.8 months). The most common all-grade adverse event was nausea (40%). The safety profile of vemurafenib was similar to that observed in melanoma studies.

**CONCLUSION:**

Vemurafenib showed promising activity in patients with NSCLC harboring *BRAF* V600 mutations. The safety profile of vemurafenib was similar to previous observations in patients with melanoma. Our results suggest a role for single-agent BRAF inhibition in patients with NSCLC and *BRAF* V600 mutations.

## INTRODUCTION

Identification of oncogenic activation of tyrosine kinases in patients with non–small-cell lung cancer (NSCLC), such as mutations in the epidermal growth factor receptor (*EGFR*) gene and rearrangements of the anaplastic lymphoma kinase (*ALK*) or *ROS1* genes, has enabled the development of targeted treatments for patients with NSCLC.^1-3^ This has resulted in the recognition of histologically and genetically diverse NSCLC subtypes and led to a targeted therapy approach for selected patients.^4^ Despite these developments, a considerable proportion of patients fail to benefit from currently available treatment regimens and need new treatment approaches.

*BRAF* V600 mutations occur in an estimated 1% to 4% of patients with NSCLC.^5,6^ Among patients with *BRAF*-mutated NSCLC, the most common aberration is the *BRAF* V600E mutation, which occurs in 50% of patients.^7^ In the melanoma setting, where *BRAF* V600 mutations are common, targeted treatment of patients with *BRAF* V600 mutation-positive metastatic melanoma using the BRAF kinase inhibitors dabrafenib and vemurafenib was associated with high response rates and improved survival compared with chemotherapy.^8-10^ Furthermore, superior outcomes were observed with dual inhibition of BRAF and MEK.^11,12^ Recently, BRAF inhibition was also shown to be effective in patients with *BRAF* V600–mutated NSCLC in a retrospective cohort study^13^ and in a clinical study of patients with *BRAF* V600E–mutated NSCLC.^14^ Dual BRAF/MEK inhibition has also been investigated as first- and second-line treatment of patients with NSCLC.^15,16^

We present the results from the expanded NSCLC cohort of the vemurafenib basket (VE-BASKET) trial. This trial assessed the efficacy of vemurafenib in seven cohorts of patients with *BRAF* V600–mutated malignancies.^17^

**CONTEXT**

**Key Objective**
To establish the efficacy and safety of vemurafenib in patients with *BRAF* V600 mutation-positive NSCLC who were enrolled in the histology-independent vemurafenib basket (VE-BASKET) trial.
**Knowledge Generated**
Vemurafenib has prolonged efficacy in patients with *BRAF* V600–mutant NSCLC (n = 62), as demonstrated by a 37% overall response rate. Response rates were similar in previously treated and untreated patients. Median progression-free survival was 6.5 months, and the median overall survival was 15.4 months; median overall survival was not reached in previously untreated patients. Clinical benefit rates for previously treated and untreated patients were 46% and 63%, respectively. No new safety signals were observed in this expanded cohort of patients with NSCLC.
**Relevance**
Single-agent vemurafenib has clinically meaningful and durable activity in patients with NSCLC harboring *BRAF* V600 mutations. This analysis adds to the overall findings of the VE-BASKET trial, which demonstrated clinically relevant activity of vemurafenib in a number of solid tumors.

## METHODS

### Study Design

The VE-BASKET study was a multicenter, single-arm, phase II study of vemurafenib in patients with a variety of nonmelanoma cancers harboring *BRAF* V600 mutations. *BRAF* V600 mutations were identified by means of mutational analysis assays routinely performed at each participating site. The clinical trial did not require central confirmation for this cohort. Six prespecified cohorts were recruited, consisting of patients with NSCLC, ovarian cancer, colorectal cancer, cholangiocarcinoma, breast cancer, and multiple myeloma; all patients with solid tumors other than those mentioned were included in a seventh cohort. Patients were treated with vemurafenib (960 mg orally two times per day) as a single agent. The design of this study has been described in detail elsewhere.^17^

This trial was performed in accordance with the provisions of the Declaration of Helsinki and Good Clinical Practice guidelines. The protocol was approved by institutional review boards or human research ethics committees at the participating centers. All patients provided written informed consent.

### Patients

Patients were eligible for inclusion in the study if they were 16 years of age or older and had histologically confirmed, measurable (Response Evaluation Criteria in Solid Tumors [RECIST], version 1.1), *BRAF* V600 mutation-positive cancers that were refractory to standard therapy or for which standard or curative therapy did not exist or was not considered appropriate by the investigator. Patients with solid tumors were required to have adequate hematologic, renal, and liver function. Patients with active or untreated CNS metastases were excluded. Prior treatment with a BRAF or MEK inhibitor was not allowed.

### Assessments

Response was assessed by the investigators according to RECIST (version 1.1). Assessments were performed using computed tomography or magnetic resonance imaging of the chest, abdomen, and pelvis at baseline and then every 8 weeks until disease progression, death, or withdrawal from the study. Adverse events (AEs) were graded by the investigators using National Cancer Institute Common Terminology Criteria for Adverse Events (version 4.0) until 28 days after discontinuation of study treatment. AEs of special interest were cutaneous squamous cell carcinoma (SCC; keratoacanthoma, squamous cell carcinoma of the skin, and Bowen disease), fatigue (fatigue and asthenia), liver injury (increased ALT, AST, blood alkaline phosphatase, blood bilirubin, and gamma-glutamyltransferase; hyperbilirubinemia, hepatocellular injury, and cholestatic jaundice), and prolonged QT interval. Patients were assessed for AEs at each clinic visit and as necessary throughout the study.

### Outcomes

The primary objective of the study was to evaluate the efficacy of vemurafenib in patients with *BRAF* V600 mutation-positive cancers. The primary end point for the final analysis in the NSCLC cohort was objective response rate (ORR), defined as the proportion of patients with an objective response (complete response [CR] or partial response [PR]) confirmed on two consecutive occasions 4 or more weeks apart. Efficacy was evaluated by the site investigators according to RECIST (version 1.1). Secondary objectives included assessments of clinical benefit rate (defined as the overall proportion of patients with a CR, PR, or stable disease lasting ≥ 6 months), duration of response, progression-free survival (PFS), overall survival (OS), and safety. Efficacy data were analyzed separately for patients who had received no prior therapy and for those with prior therapies.

### Statistical Analysis

This was a modified, two-stage Simon design study. Stage I was complete when seven patients with measurable disease were enrolled and had completed a minimum of 8 weeks of treatment, developed progressive disease, prematurely withdrew, or died. An additional six or 12 patients could be enrolled, to 13 or 19 patients, depending on the results for stage I; if two, three, or four of the initial seven patients responded to treatment, an additional 12 patients could be enrolled in stage II; if five or more of the initial seven patients responded to treatment, an additional six patients were recruited. Recruitment into any cohort/indication could be further expanded up to 70 patients if a response rate was demonstrated in stage II of that cohort, according to the stopping rules defined in the protocol or a clear clinical benefit for patients was observed, as determined by the steering committee. For the NSCLC cohort, with 50 treated patients, the study would have approximately 90% power for the lower bound of the two-sided 95% CI to exclude 20%, given a true ORR of 40%. The lower bound of the 95% CI was set at 20% because established therapy in the second and later lines had an ORR of less than 20% when the study was designed. PFS, OS, and duration of response were calculated using Kaplan-Meier methods. All analyses were performed using SAS (versions 9.2 and 9.4; SAS Institute, Cary, NC).

## RESULTS

### Patients and Treatment

A total of 62 patients with *BRAF* V600–mutated NSCLC (61 with the V600E mutation and one with an unspecified V600 mutation) were enrolled, eight (13%) of whom were previously untreated (Table 1). Most patients had adenocarcinoma (n = 58; 94%), three patients (4.8%) had CNS metastases, and most were former smokers (n = 36; 58%). Among previously treated patients, the median number of prior systemic regimens was two (interquartile range [IQR], 1 to 2); the most common prior chemotherapies were platinum agents (39 of 54 patients; 72%), pemetrexed (33 of 54 patients; 61%), and taxanes (22 of 54 patients; 41%).

**TABLE 1.**
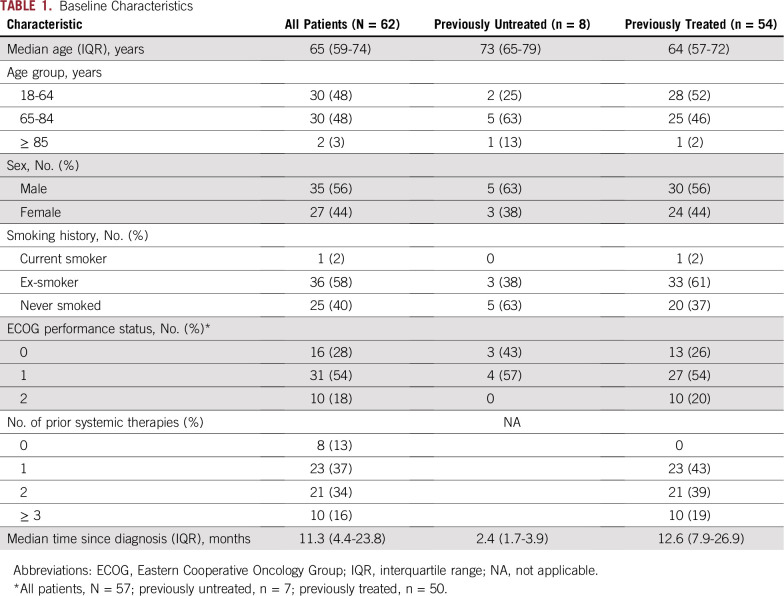
Baseline Characteristics

This analysis was performed after a median duration of follow-up of 10.7 months (IQR, 4.3 to 17.1 months). Reasons for vemurafenib discontinuation were progressive disease (41 of 62 patients; 66%), AEs (six of 62 patients; 10%), death (four of 62 patients; 6%), withdrawal by the patient (two of 62 patients; 3%), physician decision (two of 62 patients; 3%), and other reasons in the case of seven patients (11%), six (10%) of whom rolled over into an extension study and one of whom withdrew from the study.

### Efficacy

**FIG 1.**
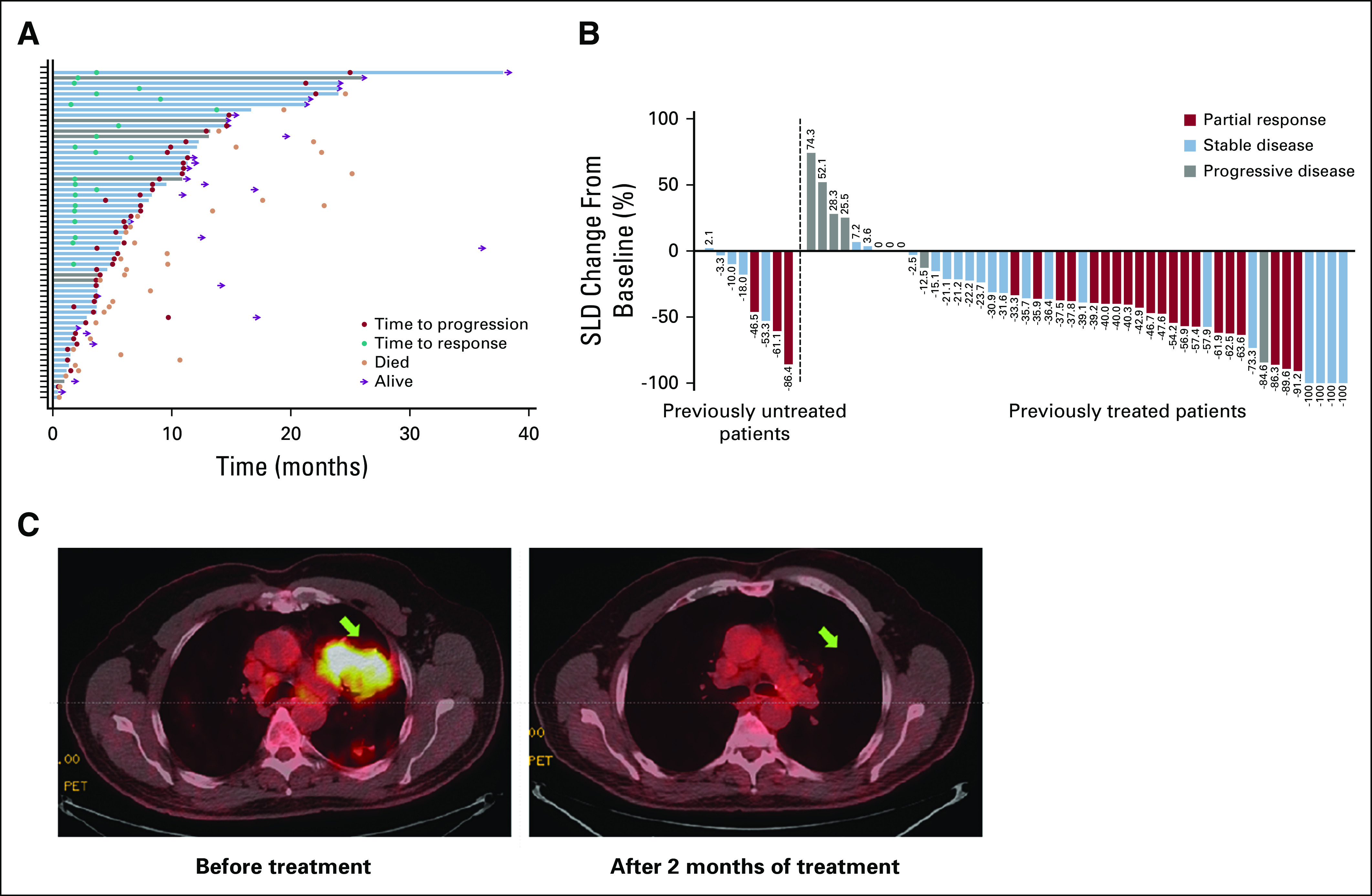
Tumor response to vemurafenib in patients with non–small-cell lung cancer (NSCLC). (A) Plot of time to progression, time to response, and death in individual patients with NSCLC. Blue bars indicate previously treated patients; gray bars indicate previously untreated patients. (B) Waterfall plot of maximum percent decrease from baseline in the sum of diameters of target tumors on the basis of investigator assessment: best overall response in individual patients. (C) Pretherapy and post-therapy ^18^F-fluorodeoxyglucose positron emission tomography images of a chemotherapy-naive patient with *BRAF* V600E mutation-positive NSCLC. The patient continues to respond to date. SLD, sum of the longest diameters.

Response to treatment is listed in Table 2 and shown in Figure 1. Overall, the investigator-determined ORR was 37% (95% CI, 25% to 50%), and the clinical benefit (CR plus PR plus stable disease lasting ≥ 6 months) rate was 48% (95% CI, 36% to 61%). Clinical benefit rates for previously treated and untreated patients were 46% (95% CI, 33% to 60%) and 63% (95% CI, 24% to 91%), respectively.

**TABLE 2.**
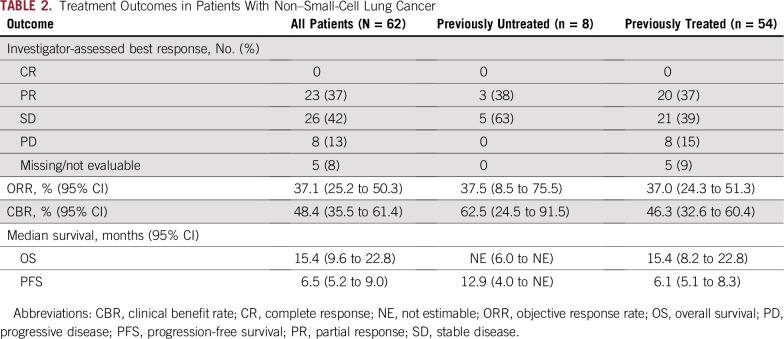
Treatment Outcomes in Patients With Non–Small-Cell Lung Cancer

The median duration of response was 7.2 months (95% CI, 5.5 to 18.4 months) in the overall population and 6.1 months (95% CI, 5.5 to 18.4 months) in previously treated patients. The median duration of response was not estimable (NE) in previously untreated patients. Median time to response was 7.3 months (95% CI, 3.7 months to NE) in the overall population and 7.3 months (95% CI, 3.7 to 13.7 months) in previously treated patients; median time to response was NE in previously untreated patients. The three previously untreated patients who responded to vemurafenib treatment had responses lasting 24.0, 7.2, and 9.1 months. At the time of study closure, there was no record of reported disease progression in six responders, including four previously treated and two previously untreated patients.

**FIG 2.**
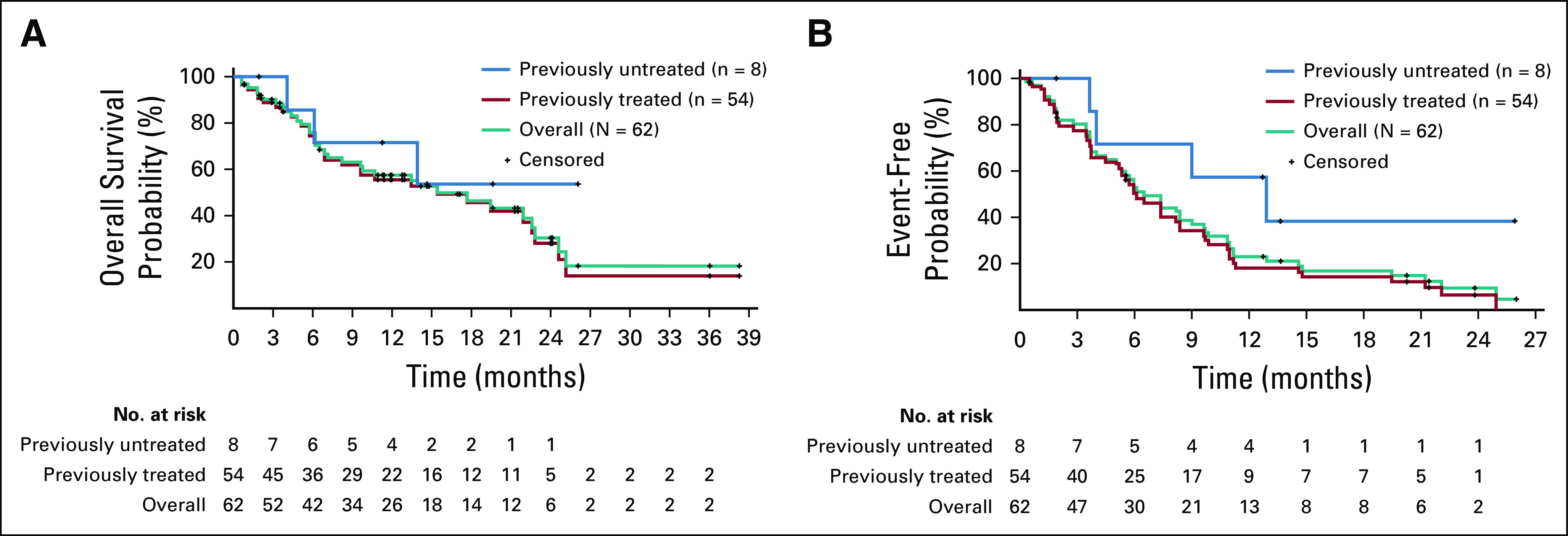
(A) Overall survival and (B) progression-free survival in patients with non–small-cell lung cancer.

The median OS was 15.4 months (95% CI, 9.6 to 22.8 months) in the overall population, 15.4 months (95% CI, 8.2 to 22.6 months) in previously treated patients, and NE in previously untreated patients (Fig 2A). OS durations in the five previously untreated patients with censored observations were 26.1, 19.6, 14.6, 11.2, and 1.9 months; OS durations were 6.0, 13.9, and 4.0 months for the three patients who had died at the time of the analysis, all of whom had a best overall response of stable disease.

Median PFS was 6.5 months (95% CI, 5.2 to 9.0 months) in the overall population and 6.1 months (95% CI, 5.1 to 8.3 months) in previously treated patients (Fig 2B). The median PFS was 12.9 months (95% CI, 4.0 months to NE) in previously untreated patients, four of whom were censored at the time of study closure (PFS: 26.0, 13.6, 1.9, and 12.7 months at study closure).

### Safety

The median treatment duration for all patients was 6.0 months (IQR, 2.8 to 11.5 months); the median treatment duration was 5.7 months (IQR, 2.8 to 11.2 months) for previously treated patients and 12.0 months (IQR, 4.0 to 13.9 months) for previously untreated patients. The median relative dose intensity achieved was 78% (IQR, 64% to 91%) overall.

All 62 patients experienced at least one any-cause AE; grade 3 or 4 AEs occurred in 48 patients (77%), and two patients had grade 5 AEs (3%; one patient with sepsis, one with a pulmonary embolism and respiratory failure; both patients had been previously treated, and none of the events were considered to be related to vemurafenib). Table 3 lists all-cause and grade 3 or greater AEs occurring in 20% or more of patients.

**TABLE 3.**
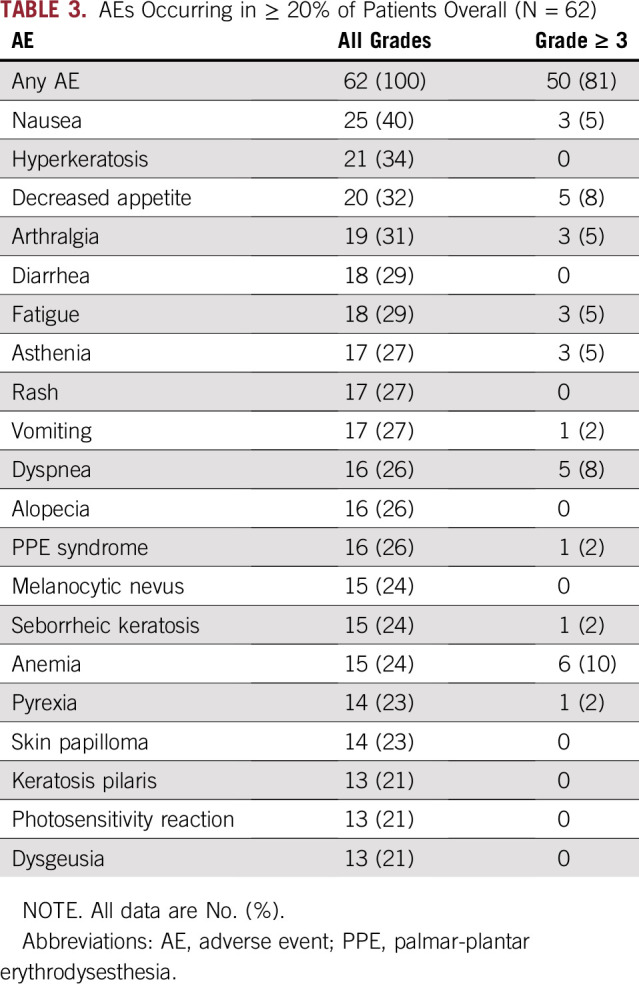
AEs Occurring in ≥ 20% of Patients Overall (N = 62)

AEs leading to treatment interruption occurred in 25 of 62 patients (40%). The most common of these were sepsis (n = 3; 5%), vomiting (n = 3; 5%), bronchitis (n = 2; 3%), pneumonia (n = 2; 3%), nausea (n = 2; 3%), acute coronary syndrome (n = 2; 3%), and dyspnea (n = 2; 3%). AEs leading to dose reduction occurred in 38 of 62 patients (61%). The most common of these events were arthralgia (n = 6; 10%), fatigue (n = 5; 8%), and decreased appetite (n = 4; 6%). Six patients had AEs that resulted in treatment discontinuation: chronic kidney disease (two of 62 patients; 3%); acute kidney injury (one of 62 patients; 2%); renal failure (one of 62 patients; 2%); lower respiratory tract infection (one of 62 patients; 2%), and oropharyngeal candidiasis and nausea (one of 62 patients; 2%).

AEs of special interest included arthralgia (19 of 62 patients; 31%), cutaneous SCC (including keratoacanthoma; 16 of 62 patients; 26%), fatigue (34 of 62 patients; 55%), prolonged QT interval (11 of 62 patients; 18%), and liver injury (increased ALT, AST, blood alkaline phosphatase, bilirubin, and gamma-glutamyltransferase, as well as the hepatobiliary disorders hyperbilirubinemia, hepatocellular injury, and cholestatic jaundice; 16 of 62 patients; 26%). A total of 82 serious AEs occurred in 39 patients (63%), the most common of which were SCC of the skin (nine patients; 15%) and keratoacanthoma (nine patients; 15%), which was defined as a serious AE. Basal cell carcinoma was observed in one patient (2%). In total, 25 patients (40%) had serious AEs considered by the investigator to be caused by vemurafenib (keratoacanthoma, n = 9; SCC of the skin, n = 9; basal cell carcinoma, n = 1; Bowen disease, n = 1; acute kidney injury, n = 4; pericarditis, n = 1; stomatitis, n = 1; pyrexia, n = 1; hypersensitivity, n = 1; sepsis, n = 1; and dehydration, n = 1); serious AEs not considered to be related to vemurafenib included pneumonia (n = 2), bronchitis (n = 2), dyspnea (n = 3), pericardial effusion (n = 1), sepsis (n = 3), pulmonary embolism (n = 2), and lung infection (n = 2).

## DISCUSSION

Targetable oncogenic drivers in NSCLC with robust clinical validation include *EGFR* mutations and *ALK* and *ROS1* fusions, but identifying other targetable, clinically important subgroups of NSCLC is a high priority. In this context, we found that patients with *BRAF* V600E–mutated NSCLC treated with vemurafenib had an ORR of 37%, with similar response rates in previously treated and untreated patients. Median OS was 15 months in the overall patient population, but had not been reached in the group of previously untreated patients after 12 months of follow-up. Similarly, our previously untreated patients had a median PFS of 12.9 months, which was considerably longer than the 6.5 months observed in patients who had received prior therapies. This may be explained either by small patient numbers or by increased acquisition of resistance mechanisms with prior therapy. This might suggest that targeted treatment in earlier lines of patients with a driver mutation could be more effective. The safety profile of vemurafenib in our group of patients with NSCLC was similar to that seen in patients with melanoma.^10,18^ No new safety signals were observed in this population. There were three patients with CNS metastases. Because response assessment in neuro-oncology–based criteria were not collected for CNS metastases, we do not have data on responses. This is one of the limitations of the study.

Our results provide evidence for the value of targeting *BRAF* with single-agent vemurafenib in patients with NSCLC. Although cross-study comparisons are made with caution, the OS we observed with single-agent vemurafenib (median, 15.4 months; 95% CI, 9.6 to 22.8 months) seems similar to that observed with the combination of dabrafenib and trametinib (median, 18.2 months; 95% CI, 14.3 months to NE), which was approved in 2017 by the US Food and Drug Administration and the European Medicines Agency for the treatment of patients with *BRAF* V600E mutation-positive NSCLC.^16^ With this approval, combination therapy consisting of a BRAF inhibitor and an MEK inhibitor has now become standard of care for patients with *BRAF* mutation-positive NSCLC, as is the case for patients with *BRAF* mutation-positive melanoma, adding to the range of targeted therapies now available for selected patients with NSCLC. We suggest that future studies should examine additional combinations in patients with *BRAF* mutation-positive NSCLC.

In conclusion, the results of the present cohort analysis suggest a role for BRAF inhibition in patients with NSCLC with *BRAF* mutations. The prolonged OS (median, 15.4 months) in the NSCLC population represents promising durability of effect with single-agent BRAF inhibition. The apparent increase in median PFS in previously untreated patients compared with previously treated patients warrants additional investigation of earlier treatment in this patient population.
